# Towards properly controlled analytical measurement methods

**DOI:** 10.1155/S1463924689000155

**Published:** 1989

**Authors:** K. M. Hangos, L. Leisztner

**Affiliations:** 1 Computer and Automation Institute Hungarian Academy of Sciences P.O. Box 63 Budapest H-1502 Hungary; 2 Institute of Forensic Science P.O. Box 314/4 Budapest H-1903 Hungary

## Abstract

It is of great practical importance to develop simple methods for the automatic detection ofthe controlled state of the analytical method being applied. The key point is to find quantities that greatly affect the quality of the analytical results and that can be easily estimated during the measurement process from the measured data. The signal-to-noise ratio has proved to be such a quantity in gas chromatographic methods. The statistical properties of the estimation of the signal-to-noise ratio from gas chromatographic data have been investigated. The suggested practical method for estimating the signal-to-noise ratio proved to be biased from a mathematical statistical point of view, but the bias is usually not greater than 10%. It has been shown by practical examples that the signal-to-noise ratio affects the quality of the analytical results and it is easy to estimate its value from practical data.


Journal of Automatic Chemistry Vol. 11, No. 2 (March–April 1989), pp. 76–79

### Towards properly controlled analytical measurement methods

**K. M. Hangos**

*Computer and Automation Institute, Hungarian Academy of Sciences, P.O. Box 63, H-1502 Budapest, Hungary*

**and L. Leisztner**

*Institute of Forensic Science, P.O. Box 314/4, H-1903 Budapest, Hungary*

*It is of great practical importance to develop simple methods for the automatic detection of the controlled state of the analytical method being applied. The key point is to find quantities that greatly affect the quality of the analytical results and that can be easily estimated during the measurement process from the measured data. The signal-to-noise ratio has proved to be such a quantity in gas chromatographic methods. The statistical properties of the estimation of the signal-to-noise ratio from gas chromatographic data have been investigated. The suggested practical method for estimating the signal-to-noise ratio proved to be biased from a mathematical statistical point of view, but the bias is usually not greater than 10%. It has been shown by practical examples that the signal-to-noise ratio affects the quality of the analytical results and it is easy to estimate its value from practical data.*

### Introduction

Highly instrumental and automated analytical measurement methods are becoming increasingly widespread. Therefore, the problems of the automated and reliable control of measurement quality are of great importance and have been thoroughly analysed in the recent literature (see, for example, Kolthoff and Elving [1] and Sharuf *et al.* [2]). It is mostly emphasized that the analytical method applied must be 'controlled', i.e., the measurement conditions must guarantee the practical absence of the systematic measurement error component and the random error component must be statistically controlled [3].

According to our previous investigations [4,5], a systematic error component can appear in the result of an analytical measurement (in the analytical information) even when only a random error component is present in the measured raw data, especially if the statistical properties of the random error can change. This effect is caused by the data processing (data reduction) step in the evaluation of the analytical measurement results and it is a consequence of the non-linearity of the data reduction transformations. These transformations and hence the resulting systematic error component depend on the true value of the raw measured data, and also on the characteristics (type and variance) of the random measurement error component and the algorithm used.

The above difficulties can be overcome by suitable control of the measurement conditions affecting the parameters of the random error component which are constant during the whole measurement procedure. However, in this instance, a need to control the properties and especially the relative standard deviation (RSD) of the random error component arises. We showed previously [6] by applying Monte Carlo methods that it is possible to estimate the standard deviation of the measured data using the statistics of the difference test. Our long-term practical experience has proved the high practical value of such control.

The aim of this work was to investigate the methods of estimation and control of the measurement conditions affecting the data reduction and to establish the practical usefulness of the proposed method.

### Concept of the signal-to-noise ratio

The signal-to-noise ratio [7] proved to be a very useful concept in characterizing the measurement conditions with respect to the measurement error. This part of the paper is devoted to the definition of the signal-to-noise ratio.

Let us model the sequence of the raw measurement signals in the form

$$y_i = \sum_{j=1}^{K} f_{ji} + b_i + \varepsilon_i \qquad (i = 1, \ldots, N) \qquad (1)$$

where

$y_i$ = element of the raw measured value sequence;
$b_i$ = element of the baseline sequence;
$\varepsilon_i$ = element of the random measurement error sequence;
$K$ = number of chemical compounds to be measured;
$N$ = number of measured signal values;
$f_{ij}$ = discrete deterministic function (sequence) which describes the ideal measurement sequence for the $j$th compound (describes a single ideal peak).

The above model describes the sequence of the raw measurement signals for instrumental analytical methods with probability density function-type signals. Note that the outliers have been omitted from the model because they can easily be filtered out from the raw measurement signal sequence. Their frequent presence indicates that the measurement conditions are unsatisfactory.

The signal-to-noise ratio with respect to the $j$th compound can be defined as

$$E = \frac{H_j}{s} \qquad (2)$$

0142–0453/89 $3.00 © 1989 Taylor & Francis Ltd.

where

$E$ = signal-to-noise ratio;
$H_j$ = peak height of the $j$th compound;
$s$ = standard deviation (square root of the variance) of the random measurement error component in place of the peak height.

Note that the above definition implies that the mean value of the random measurement component is zero. It is also interesting that the signal-to-noise ratio is the reciprocal of the relative standard deviation of the peak height.

It is important to note that the type of probability density of the random measurement error component (whether it is normally or uniformly distributed) does not influence the data reduction, in our experience [4, 5]. Hence it is sufficient to use its standard deviation in equation (2).

The standard deviation of the random measurement error component may vary with the mean value of the raw measurement signal, i.e., also with the value of peak height. There is also a real danger that the standard deviation varies with $i$, in this instance with the retention time. The first fact causes difficulties in estimating the signal-to-noise ratio from the data of a single measurement sequence, and the second may require the control of the signal-to-noise ratio in several retention time intervals. The solution to these problems lies in the special nature of the data reduction for our case and in the requirements for the measurement control, and will be discussed later.

The true sequence of the raw measured value sequence can be defined as follows:

$$\hat{y}_i = y_i - \varepsilon_i = \sum_{j=1}^{K} f_{ji} + b_i \qquad (3)$$

### Estimation of the signal-to-noise ratio

If the need to estimate the signal-to-noise ratio defined by equation (2) arises from a single sequence of raw measured signals, serious theoretical difficulties arise. As the peak height is represented in only a single element of this sequence, say in $y_H$, there is no 'sample' in the sense of mathematical statistics for which elements would represent the same random variable with a definite probability density function. In ergodic (in our case stationary) stochastic processes, this difficulty can be overcome by using a subset from the sequence as a sample instead of measuring a sample from the measurement sequences. However, with peak height, no subset of the raw measurement signal sequences will form a sample representing the probability distribution in the peak height. Further, repeated measurements with unknown and varying peak heights give no further information about the signal-to-noise ratio, as the actual value of the signal-to-noise ratio in this instance depends not only on the variations in the measurement conditons but also on the variation of the peak height.

For the above reasons, only approximate estimates of the signal-to-noise ratio can be calculated from a single measured value sequence. One possible estimate has been proposed by Leisztner and Barna [6] which fits the usual evaluation (data reduction) methods very well. This estimation procedure constructs the peak height and the variance of the random measurement error component separately based on a subset of the whole raw measured data sequence belonging to the $j$th peak. This subset is formed by the so-called peak detection algorithm in the data reduction (for a detailed description, see Hangos and Leisztner [5]).

*Estimation of the peak height*

The peak height is estimated by the usual peak height determination algorithm in the data reduction, which fits a second-order polynomial to the data near to the peak height (let us denote the number of their members by $n_p$) and the estimated value of the peak height is estimated as the maximum of this polynomial. It is easy to see that this procedure gives a biased estimate of the peak height even if the distribution of the random measurement error component is Gaussian, because the peaks do not have the shape of a second-order polynomial; their shape may vary and cannot be described by a general mathematical equation (in most instances they have a nearly Gaussian shape). However, the estimated peak height can be regarded approximately as a random variable with a Gaussian distribution.

*Estimation of the standard deviation of the random measurement error component in the peak height*

This estimation is based on the following assumptions: (1) the distribution (and hence the standard deviation) of the random measurement error component remains unchanged throughout the peak and does not vary with the true value of the raw measured data samples (note that this assumption is only approximately valid); and (2) the sampling rate is high enough to have small enough changes in the true value sequence to approximate these changes by a linear relationship for each of three consecutive changes (note that this assumption cannot be valid near to the peak height and near to the beginning or end of the peak because the second derivative of the peak shape is large in these regions).

Hence the required standard deviation can be estimated from a subset of the samples belonging to the $j$th peak satisfying assumption (2). Let us denote their number by $n_m$. The true value of the raw measured data from this subset is estimated by its smoothed value:

$$\bar{y}_i = \frac{y_{i+1} + y_i + y_{i-1}}{3} \qquad (4)$$

Note that this estimate is biased and the magnitude of the bias depends on the value $y''_i \Delta t$, where $y''_i$ is the value of the second derivative of the true value function and $\Delta t$ is the sampling interval. If the random measurement error component is normally distributed, then the above estimate is also normally distributed with variance $s^2/3$ according to assumption (1).

Note that in practical situations the Gaussian distribution of the random measurement error component cannot be assumed, but in the presence of independent noise sources in the limit distribution should generally be Gaussian according to the central limit theorem. Unfortunately, there are significant sensors in instrumental analytical chemistry with highly non-Gaussian (uniform) noise, so the very useful assumption about a Gaussian distribution of the random measurement component must be treated with caution.

Using the above estimate of the true value sequence, the variance can be estimated by the equation.

$$\bar{s}^2 = \frac{\sum_{i=1}^{n_m} (\bar{y}_i - y_i - \bar{y}_{i+1} + y_{i+1})^2}{2(n_m - 1)} \qquad (5)$$

The squared terms in equation (5) can be expressed as the estimated values of the random measurement error components, $\varepsilon_i - \varepsilon_{i+1}$, and therefore $\bar{s}^2$ is identical with the statistics applied to the variance in a difference test [8].

In the special case of normally distributed random error components, the estimate $s^2$ has an approximately $\chi^2$ distribution, neglecting the variance of the estimate $\bar{y}_i$ compared with the variance $s^2$.

It is important to note that with an approximately linear mean value of the measured data sequence (in time, i.e., in $i$) during the interval $(i - 1, i + 2)$, the above statistics provide a suitable drift elimination. This is easy to see if one expands one term in the denominator of equation (5) using the definition in equation (4):

$$E(y_i - \bar{y}_i + y_{i+1} + \bar{y}_{i+1}) = \frac{1}{3} E[2(y_i - y_{i+1}) - (y_{i-1} - y_i) - (y_{i+1} - y_{i+2})] \approx 0 \qquad (6)$$

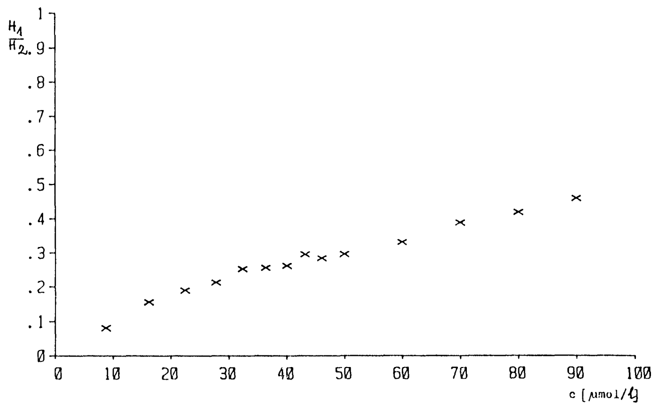

*Figure 1. Calibration for determination of acetaldehyde with a sample holder volume of 22 ml. $H_1$ = acetaldehyde peak height; $H_2$ = propan-1-ol (internal standard) peak height; c = concentration (μmol/l).*

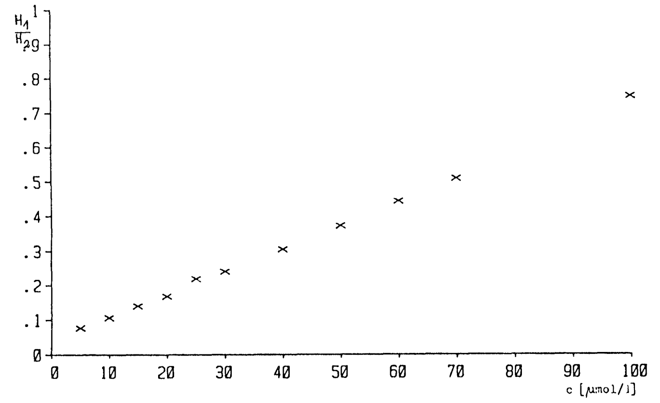

*Figure 2. Calibration for determination of acetaldehyde with a sample holder volume of 5 ml. $H_1$ = acetaldehyde peak height; $H_2$ = propan-1-ol (internal standard) peak height; c = concentration (μmol/l).*

### Possibilities of correct measurement control

In spite of the fact that the estimation of the signal-to-noise ratio is not an easy task, this quantity proved useful in characterizing the measurement conditions affecting the data reduction transformation even for peaks of unknown components with varying heights. This seems surprising because the peak height may vary over a wide range for unknown components, while the standard deviation remains approximately unchanged. The answer to this contradiction lies in the fact that the peak shape can be regarded as unchanged in a restricted range as its height increases. Hence the relative error in the peak determination increases with decreasing peak height.

From the viewpoint of measurement control it is advantageous, however, that there exists a lower limit for the signal-to-noise ratio above which the systematic error component caused by random errors is negligible. Therefore, it is often sufficient to check whether the signal-to-noise ratio is above this limit.

In order to implement a correct measurement control system from the viewpoint of data reduction, the suggested steps are as follows.

### *In the calibration phase*

Together with the estimation of the parameters of the calibration graph, the determination of the lower limit (or if the measurement conditions do not permit measurements above this limit, then a precise value) of the signal-to-noise ratio is required above which (or under which) the calibration is valid. Figures 1 and 2 show the variation of the calibration graph with the signal-to-noise ratio. These calibrations were used by us for the determination of acetaldehyde by headspace gas chromatography using propan-1-ol as internal standard (for the analytical details, see Nagy *et al.* [9]). The calibration shown in figure 1 has an S-shape. Having increased the signal-to-noise ratio 5-fold (the method used was described by Leisztner *et al.* [10]), we obtained a linear relationship as demonstrated in figure 2. The signal-to-noise ratio belonging to figure 2 can be regarded as the lower limit value.

*Table 1. Results of solution with 70 μmol/l acetaldehyde concentration with propan-1-ol as internal standard*

| No. | Peak area | Peak area ratio | Peak height | $\bar{s}$ | Signal-to-noise ratio |
|---|---|---|---|---|---|
| 1 | 1696·17 | 0·065 | 213·033 | 0·7727 | 275·696 |
| 2 | 1709·33 | 0·0672 | 218·831 | 0·7949 | 275·26 |
| 3 | 1749·33 | 0·0677 | 224·564 | 0·7695 | 291·818 |
| 4 | 1515·83 | 0·0592 | 221·093 | 4·0676 | 54·3541 |
| 5 | 1717·98 | 0·0683 | 219·879 | 0·7988 | 275·26 |
| Mean | 1677·73 | 0·0655 | 219·48 | 1·44074 | 234·478 |
| Standard deviation | 92·59 | 3·71 × $10^{-3}$ | 4·2 | 1·46 | 100·94 |
| Relative standard deviation (%) | 5·51 | 5·69 | 1·91 | 101·92 | 43·05 |
| Corrected mean | 1718·2 | 0·0671 | 219·08 | 0·7839 | 279·51 |
| Corrected standard deviation | 22·61 | 1·4 × $10^{-3}$ | 4·74 | 1·5 × $10^{-2}$ | 8·21 |
| Corrected relative standard deviation (%) | 1·32 | 2·15 | 2·16 | 1·91 | 2·94 |

In this phase, when repeated measurements with known samples are available, it is possible to check whether the signal-to-noise ratio depends on the $i$ time index. If it does then it must be investigated how this dependence varies with variations in the measurement conditions.

*In the measurement control phase*

It is necessary to estimate the signal-to-noise ratio on-line during the whole measurement process. The comparison of the estimated signal-to-noise values with the desired value resulting in the first step above can be approximately performed with a one-sided (when a lower limit of the signal-to-noise ratio exists) or with an ordinary $t$-test for two samples [11].

It is important to note that the bias or the systematic error component depends very weakly on the signal-to-noise ratio, hence we are interested only in a rough estimate of the signal-to-noise ratio for measurement control purposes. Therefore, the exact distribution of the statistics estimating the signal-to-noise ratio does not have great significance from a practical point of view.

Table 1 shows the results of five repeated determinations of acetaldehyde by headspace gas chromatography using propan-1-ol as internal standard. The evaluation of the measurement results was two-fold, from the peak area ratio (ratio of the peak area of acetaldehyde to that of the internal standard) and the peak height ratio. The signal-to-noise ratio in the fourth determination was approximately six times smaller than that in the others (for further details, see Nagy *et al.* [9]). We obtained a better mean with a smaller standard deviation if we omitted this outlying result, as is shown by the corrected values in table 1.

### Conclusion

The proposed practical method for estimating the signal-to-noise ratio from real chromatographic data proved to be biased from a theoretical point of view. However, it is very useful for measurement control purposes and the bias in the estimate of the signal-to-noise ratio is usually not greater than 10%.

### References

1. Kolthoff, I. M. and Elving, P. J. (Ed.), *Treatise on Analytical Chemistry, Part I.* (Wiley, New York, 1978).
2. Sharuf, H. A., Illman, D. L., and Kowalski, B. R., *Chemometrics* (Wiley, New York, 1987).
3. De Voe, J. R. (Ed.) *Validation of the Measurement Process* (ACS Symposium Series, American Chemical Society, Washington, DC, 1977).
4. Leisztner, L., Kuzmin, N. M., and Barna, P., *Zhurnal Analiticheskoi Khimii,* **38** (1983), 2247.
5. Hangos, K. M., and Leisztner, L., *Journal of Automatic Chemistry,* **9** (1987), 25.
6. Leisztner, L. and Barna, P., *Journal of Automatic Chemistry,* **5** (1983), 155.
7. Smit, H. C., and Walk, H. L., *Chromatographia,* **8** (1975), 311.
8. Hart, B. I., *Annals of Mathematical Statistics,* **13** (1942), 445.
9. Nagy, J. L., Leisztner, L., and Véchelyi, P. V., *Analytical Biochemistry,* **151** (1985), 365.
10. Leisztner, L., Nagy, J. L., and Ulrich, E. *Journal of High Resolution Chromatography and Chromatography Communications,* **5** (1982), 48.
11. Rao, C. R., *Linear Statistical Inference and its Application* (Wiley, New York, 1973).

